# Metal-Free Solvent Promoted Oxidation of Benzylic Secondary Amines to Nitrones with H_2_O_2_

**DOI:** 10.1021/acs.joc.1c01888

**Published:** 2021-09-16

**Authors:** Álisson Silva Granato, Giovanni Wilson Amarante, Javier Adrio

**Affiliations:** † Departamento de Química Orgánica, Facultad de Ciencias, Universidad Autónoma de Madrid, Cantoblanco, 28049 Madrid, Spain; ‡ Institute for Advanced Research in Chemical Sciences (IAdChem), Universidad Autónoma de Madrid, 28049 Madrid, Spain; § Chemistry Department, Federal University of Juiz de Fora, Sao Pedro, Juiz de Fora 36036-900, Brazil

## Abstract

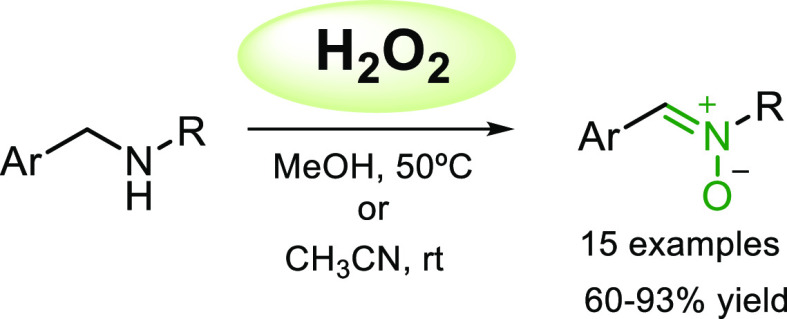

An environmentally benign protocol for the generation of nitrones from benzylic secondary amines via catalyst-free oxidation of secondary amines using H_2_O_2_ in MeOH or CH_3_CN is described. This methodology provides a selective access to a variety of C-aryl nitrones in yields of 60 to 93%. Several studies have been performed to shed light on the reaction mechanism and the role of the solvent.

The development of highly efficient and environmentally friendly methodologies for the preparation of nitrones is of great importance since this kind of compounds are valuable synthetic intermediates and useful scaffolds in drug discovery.^1^ Nitrones are found in numerous natural products^2^ and many studies have demonstrated the interest of benzylic nitrones as therapeutic agents for several pathologies including atherosclerosis, septicaemia, stroke, and Alzheimer^3^ (Figure 1).

**Figure 1:**
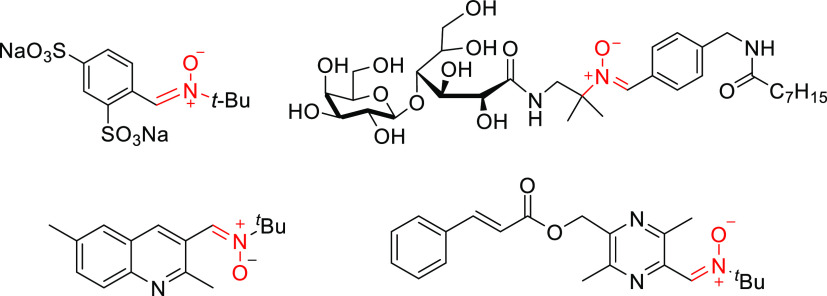
Examples of biologically active nitrones.

In addition, nitrones have been used as ligands in organometallic chemistry^4^ and as spin traps in biological studies.^5^ The diastereo- and enantioselective nucleophilic additions to nitrones is a fundamental tool in organic synthesis.^6^ Furthermore, the 1,3-dipolar cycloaddition reaction of nitrones with alkenes has become one of the methods of choice for the preparation of isoxazolidines, and a wide variety of natural products has been prepared using this reaction as key step.^7^

Among the available methodologies for the preparation of nitrones, the condensation of carbonyl compounds with hydroxylamines has arguably been the most used.^8^ However, this procedure presents several limitations, such as the availability of the hydroxylamines and the low reactivity observed using ketones as carbonyl partner. Otherwise, nitro compounds can be used as an alternative to hydroxylamines under reductive conditions.^9^

Another main process for the synthesis of nitrones consists of the oxidation of secondary hydroxylamines,^10^ imines (either preformed^11^ or generated in situ form primary amines and aldehydes^12^), isoxazolidines,^13^*N*-alkyl-α-amino acids^14^ (via regioselective decarboxylative oxidation), and secondary amines. The great availability of secondary amines has made the last option one of the most convenient. In fact, this methodology has been used for preparative scale and as key step in the synthesis of several natural products.^15^

Among all the oxidants used for nitrone synthesis from amines, hydrogen peroxide is one of the most attractive for the development of environmentally friendly processes since water is the only by product of its reduction. In 1984, Murahashi and co-workers reported the first example of catalytic direct oxidation of secondary amines to nitrones with H_2_O_2_ in the presence of Na_2_WO_4_/H_2_O.^16^ Since then, several general and efficient procedures have been developed using hydrogen peroxide or its urea complex (UHP) as oxidant in combination with different catalysts such as SeO_2_,^17^ methyltrioxorhenium,^18^ and titanium^19^ or platinum^20^ complexes. In addition, several heterogeneous catalysts have also been used.^21^ Alternatively, the reaction can be also carried out in the presence of alkyl hydroperoxides,^22^ oxone,^23^ dimethyldioxirane,^24^*m*-CPBA,^25^ Davis oxaziridine,^26^ or molecular oxygen^27^ as oxidant.

To the best of our knowledge, most of the procedures described to date for the preparation of nitrones from amines using H_2_O_2_ as oxidant require the presence of a metal catalyst, which is usually expensive and present environmental problems. Herein, we report a facile and clean catalyst-free oxidation protocol for the efficient preparation of nitrones from benzylic secondary amines using hydrogen peroxide as oxidant.

1,2,3,4-Tetrahydroisoquinoline was selected as the model substrate for the oxidation process. This substrate is useful for comparative purposes since its oxidation to nitrone with the combination of different oxidants and catalytic systems has been extensively studied.^1^ Initially, the reaction was performed in the presence of four equivalents of H_2_O_2_ 30% v/v in MeOH at room temperature, leading to the formation of the nitrone with a 27% of conversion after 24 h (Table 1, entry 1). An increase of the equivalents of hydrogen peroxide leads to complete conversion (entries 2 and 3). Higher reactivity was observed at 50 °C allowing reducing the amount of H_2_O_2_ from 10 to 4 equiv and the reaction time to 12 h (entry 4). Further decreasing the amount of hydrogen peroxide revealed that the reaction proceeded with a significant erosion of the reactivity (entries 5 and 6). Similar results were obtained using EtOH as solvent (entry 7). On the other hand, using less polar aprotic solvents, such as CH_2_Cl_2_ or toluene, no conversion was observed (entries 8 and 9). Interestingly, a very significant improvement of the reactivity was observed using CH_3_CN as solvent, complete conversion was achieved in only 2 h at room temperature (entry 10). A similar outcome was observed using only 2 equiv of H_2_O_2_ (entry 11). Finally, no reaction was observed using a solvent with similar dielectric constant such a DMF (entry 12).

**Table 1:**
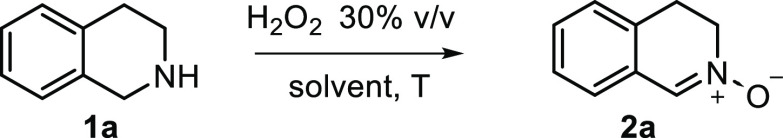
Optimization of the Reaction Conditions

entry	equiv	*T* (°C)	solvent	time (h)	conversion (%)^a^
1	4	rt	MeOH	24	27^c^
2	8	rt	MeOH	24	60
3	10	rt	MeOH	24	>99
4	4	50	MeOH	12	>99 (91)^b^
5	2	50	MeOH	12	45^c^
6	3	50	MeOH	12	73^c^
7	4	50	EtOH	12	>99
8	4	50	CH_2_Cl_2_	24	
9	4	50	toluene	24	
10	4	rt	CH_3_CN	2	>99 (93)^b^
11	2	rt	CH_3_CN	2	>99 (90)^b^
12	2	rt	DMF	12	

a Determined by ^1^H NMR in the crude reaction mixture.

b Yield after chromatographic purification.

c Nitrone is the only observed product in the ^1^H NMR spectra.

With the optimized reaction conditions on hands, we then investigated the scope of this oxidation reaction (Table 2). A remarkably broad range of benzylic secondary amines could be converted into the corresponding nitrones with good yields using either MeOH (conditions A) or CH_3_CN (conditions B) as solvent.

**Table 2:**
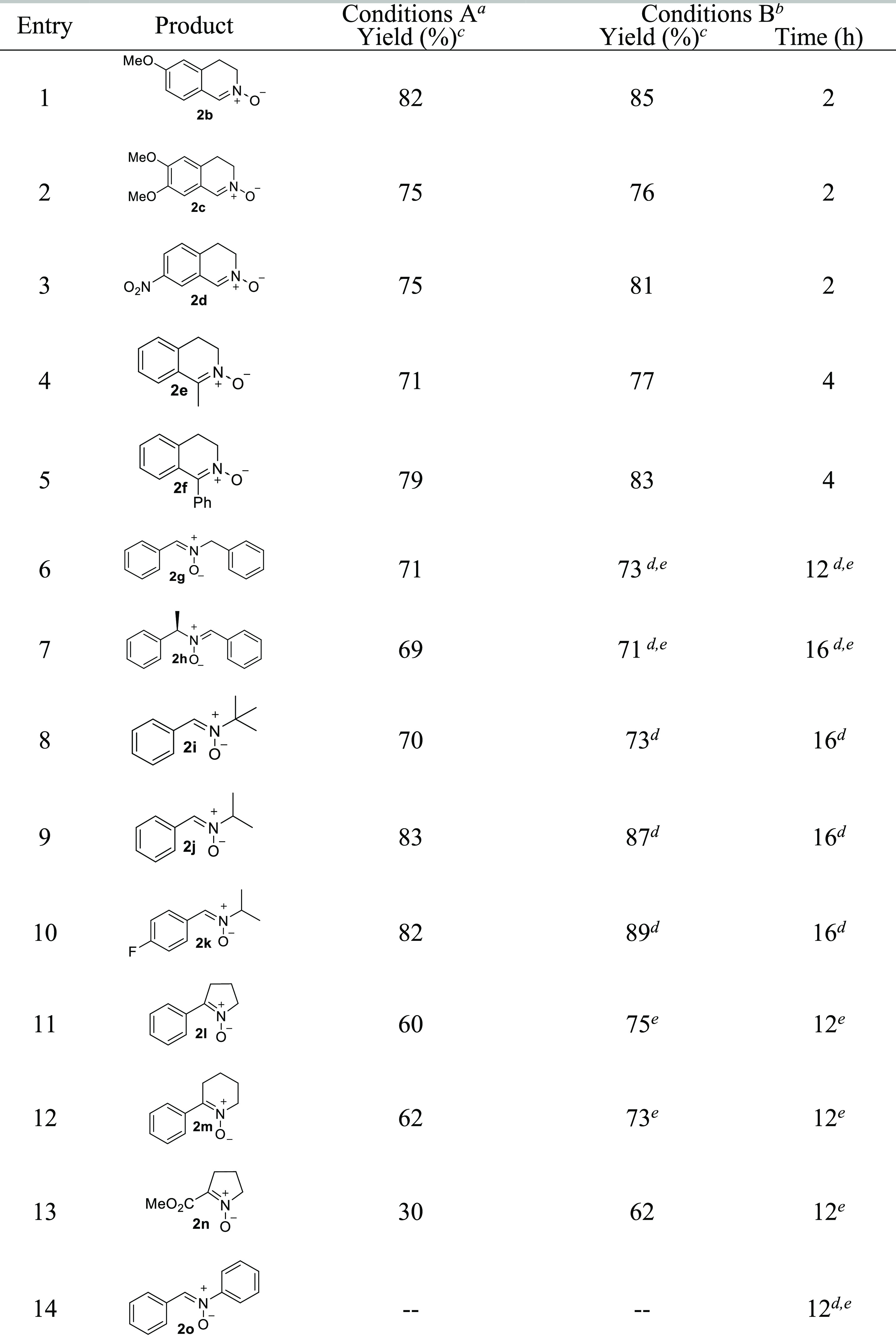
Scope of Oxidation of Benzylic Secondary Amines to Nitrones

a Conditions A: H_2_O_2_ 30% v/v (4 equiv), MeOH, 50 °C, 12 h.

b Conditions B: H_2_O_2_ 30% v/v (2 equiv), CH_3_CN, rt.

c Yield after chromatographic purification.

d Reaction at 50 °C.

e 4 equiv of H_2_O_2_ is used.

In general, better reactivity and slightly superior yields were observed using CH_3_CN as solvent in most of the examples studied. The influence of electronic character of the substituents was first evaluated. Tetrahydroisoquinolines **1b** and **1c** with strong electron donating substituents like methoxy afforded the corresponding nitrones with high yields both using conditions A or B (Table 2, entries 1 and 2). The reaction also tolerates electron withdrawing groups. For instance, 6-nitrotetrahydroisoquinoline effectively provides the desired nitrone **2d** in high yield (75% conditions A or 81% conditions B; entry 3). 1,2,3,4-Tetrahydroisoquinolines with alkyl (**2e**) or aryl (**2f**) substituents at position 1 were selectively oxidized in the benzylic more substituted position to the corresponding nitrone derivatives under both conditions (Table 2, entries 4 and 5). Dibenzylamine **1g** was cleanly converted to nitrone **2g** in 71% (MeOH) or 73% (CH_3_CN) yield(entry 6). Interestingly, this methodology also allowed the straightforward preparation of chiral nitrone **2h**,^28^ which has been extensively employed in diastereoselective 1,3-dipolar cycloadditions (entry 7). In this example, under both conditions, the reaction takes place in the less hindered site, suggesting kinetic control. Acyclic *N*-benzyl-*N*-alkyl substituted amines **1i,j,k** were selectively oxidized only on benzyl position to nitrones **2i,j,k** in good yields (entries 8, 9, and 10), although it is required to carry out the reaction at 50 °C in both solvents. The oxidation of 2-phenylpyrrolidine **1l** and 2-phenylpiperidine **1m** also proceeded efficiently, leading to nitrones **2l** and **2m** in comparable yields (entries 11 and 12). Benzylic secondary amine **1n** was also a suitable substrate, albeit the process occurred with a somewhat lower yield. No formation of nitrone **2o** was observed when less nucleophilic *N*-benzylaniline was tested under the same reaction conditions and most of the starting material was recovered unaltered. Dialkylamines are not suitable substrates for this transformation, the reaction did not occur with cyclic (piperidine) or acyclic (dioctylamine) substrates. In these examples, complex reaction mixtures were obtained under optimized reaction condition using MeOH or CH_3_CN as solvent.

To demonstrate the robustness and the synthetic utility of the method, we scaled up the oxidation reaction either in CH_3_CN or MeOH using 15 mmol of tetrahydroisoquinoline **1**. In both cases, the desired nitrone **2** was isolated in excellent yields (Scheme 1, eq A). The reaction can also be carried out using the urea-hydrogen peroxide adduct (UHP), a safe source of hydrogen peroxide. UHP is cheap, easy to handle, and can be stored for long periods without any change of the oxygen content^29,11a^ (Scheme 1, eq B).

**Scheme 1:**
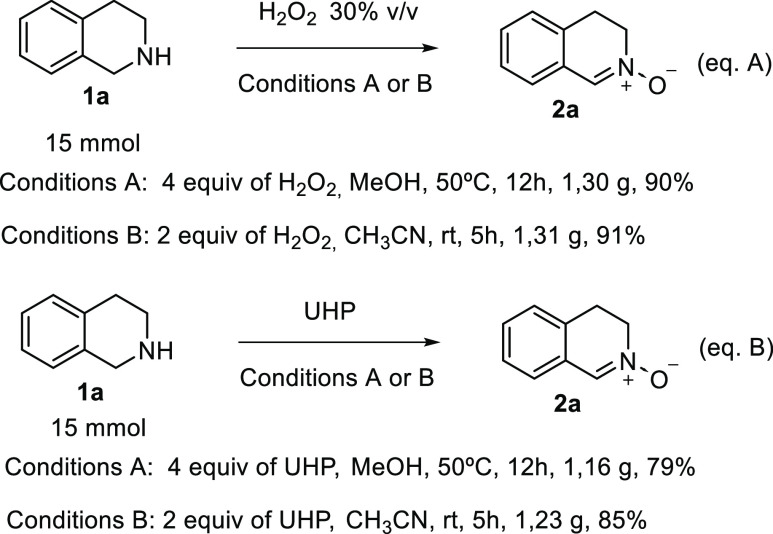
Scale-up of the Oxidation of **1a** and Use of UHP as the Source of Hydrogen Peroxide

Hydrogen peroxide has been extensively used as primary oxidant in tertiary amine oxidations under either heterogeneous or homogeneous catalytic conditions.^20^ Nevertheless, the reaction of tertiary amine **3** or electron richer trialkylamine **4** with H_2_O_2_ in MeOH at 50 °C did not show the *N*-oxide formation (Scheme 2, eq A). Taking advantage of this chemoselectivity, a secondary amine could be selectively oxidized to nitrone in the presence of a tertiary amine. Thus, oxidation of tetrahydroisoquinoline **1o** exclusively afforded nitrone **2o** in 79% yield (Scheme 2, eq B).

**Scheme 2:**
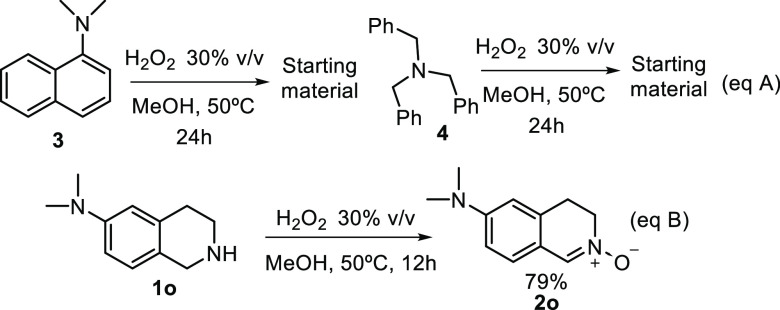
Selective Oxidation of Secondary Amines in the Presence of Tertiary Amines

Next, to gain some insights into the reaction mechanism some experiments were performed. It is well stablished that hydrogen peroxide could be activated toward nucleophilic attack by the formation of a hydrogen bond.^30^ We hypothesized that H_2_O_2_ could be electrophilically activated by MeOH, the OH moiety of the solvent forms a hydrogen bond with H_2_O_2_ increasing the electrophilic character of the oxygen. Accordingly, H_2_O_2_ did not oxidize secondary amines in aprotic solvents such as CH_2_Cl_2_ or toluene. However, using UHP as hydrogen peroxide source the reaction can be performed in toluene probably because the urea is able to activate H_2_O_2_ by hydrogen bonding formation. Furthermore, reactions in hexafluoro 2-propanol (HFIP) are faster than in MeOH since the hydroxyl proton of HFIP forms a stronger hydrogen bond because of the electron-withdrawing character of CF_3_ group.^31^ Interestingly, the use of HFIP as solvent allowed the reduction of the number of equivalents of hydrogen peroxide from 4 to 2 without erosion in reactivity. However, only 40% of conversion was observed when the reaction was performed at room temperature (Scheme 3).

**Scheme 3:**
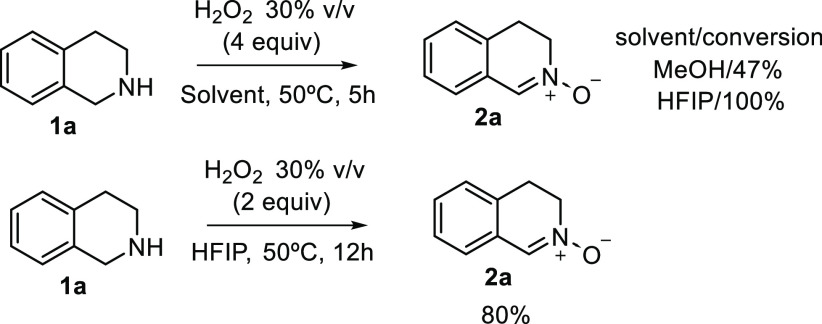
Reaction Using HFIP as Solvent

On the other hand, it has been reported that the rate of several oxidation reactions using aqueous H_2_O_2_ is significatively increased in the presence of a nitrile in basic media via the formation of a peroxymidic acid intermediate which rapidly reacts with the secondary amine to afford the corresponding nitrone and acetamide.^32^ The oxidation of **1a** in acetonitrile as solvent was monitored by ESI-MS detecting small amounts of the ion [M + H^+^] (60.0446 *m*/*z*) that correspond to acetamide. This oxidation process provides similar results using only 2.5 equiv of acetonitrile or 4-bromobenzonitrile in toluene as solvent (Scheme 4, eq A). However, only 5% of conversion of the 4-bromobenzonitrile into the 4-bromophenylacetamide was observed in the crude ^1^H NMR. In addition, 4-bromobenzonitrile was recovered unaltered after 12 h of reaction with 2 equiv of H_2_O_2_ in toluene at room temperature (Scheme 4, eq B). These results suggest that the peroxyacetimidic acid is not the major oxidizing species in these reactions.

**Scheme 4:**
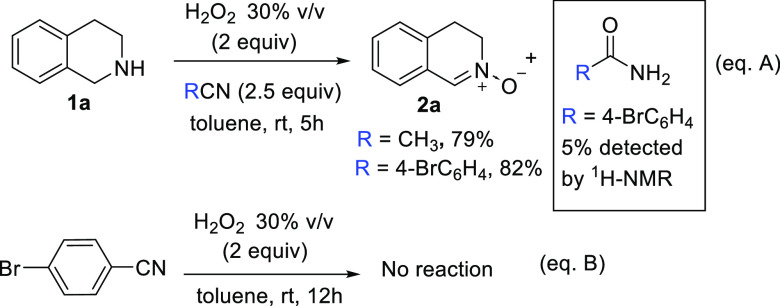
ESI-MS Experiment and Reaction with 2.5 equiv of Nitrile

It has been proposed that the oxidation of secondary amines to nitrones is a two-step sequence involving an initial formation of a hydroxylamine followed by oxidation of the latter to nitrone.^20^ Alternatively, nitrones can also be prepared by oxidation of imines.^11,12^ In our case, the reaction of imine **5** under the optimized oxidation conditions did not give the corresponding nitrone, recovering the starting material together with degradation products. On the other hand, the oxidation of commercially available dibenzylhydroxylamine **6** took place with complete conversion to the corresponding nitrone **2g**. These results suggested that the hydroxylamine and not the imine is the intermediate in the reaction pathway (Scheme 5).

**Scheme 5:**
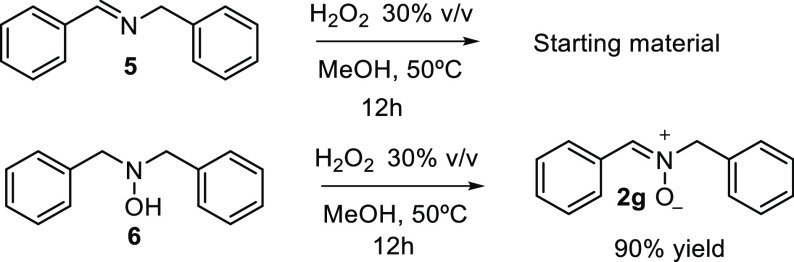
Control Experiments

As mentioned before, 1,3-dipolar cycloaddition of nitrones is one of the most straightforward methodologies for the preparation of isoxazolidines. We next studied the possibility of carrying out a one pot 1,3-dipolar cycloaddition of the obtained nitrones with alkenes. Tetrahydroquinoline **1a** was treated with H_2_O_2_ in MeOH at 50 °C for 12h, subsequent addition of *N*-methyl or *N*-phenyl maleimide to the reaction mixture afforded the corresponding cycloadduct *exo*-**7** or *exo*-**8**, as a single diastereomer, in high yield, after 4 h.^33^ (Scheme 6).

**Scheme 6:**
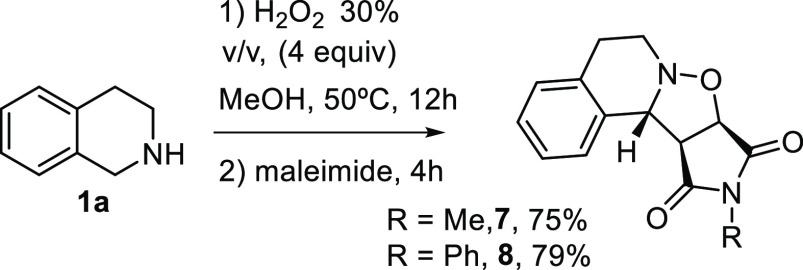
1,3-Dipolar Cycloaddition

We have developed a novel procedure for the selective oxidation of benzylic secondary amines to nitrones using H_2_O_2_ as the sole oxidant in MeOH or CH_3_CN. An important advantage of this methodology is that the reaction can be performed under mild reaction conditions without any catalyst or additive. It is also possible to carry out the reaction using UHP as a safe source of anhydrous hydrogen peroxide. Remarkably, the system allows the selective oxidation of secondary amines in the presence of tertiary amines. Several studies were performed in order to shed light on the ability of MeOH and CH_3_CN to activate H_2_O_2_.

## Experimental Section

### General Methods

Dichloromethane and toluene were dried over the PureSolv MD purification system. Reactions were monitored by thin-layer chromatography carried out on 0.25 mm silica gel plates (230–400 mesh). Flash column chromatographies were performed using silica gel (230–400 mesh). NMR spectra were recorded on AU-300 MHz instrument and calibrated using residual undeuterated solvent (CDCl_3_) as internal reference. MS spectra were recorded on a VG *AutoSpec* mass spectrometer.

All the chromatographic columns were carried out using deactivated silica gel. *Deactivated silica gel preparation*: Et_3_N (5 mL) was added to a suspension of 300g of silica gel in cyclohexane; the mixture was stirred for 1 h, filtered, and dried under reduced pressure on a rotary evaporator.

Nitrones **2a**,^23^**2b**,^34^**2c**,^35^**2d**,^36^**2e**,^37^**2f**,^38^**2g**,^23^**2h**,^39^**2i**,^40^**2j**,^41^**2l**,^42^**2m**,^43^ and **2n**(42) and cycloadducts **6** and **7**(33) have been previously described. Spectroscopic data match those previously reported.

### General Procedure 1 (Conditions A)

To a stirred solution of amine (1 mmol) in MeOH (3 mL) H_2_O_2_ 30% v/v (4 mmol, 453 μL) was added. The resulting solution was stirred at 50 °C (oil bath) for 12 h, after cooling at room temperature CH_2_Cl_2_ (10 mL) and water (10 mL) were added. The organic layer was separated, and the aqueous phase was extracted with dichloromethane (10 mL). The combined organic layers were washed with brine, dried over MgSO_4_, and evaporated under reduced pressure. The crude mixture was purified by flash column chromatography over deactivated silica gel to afford the corresponding nitrone.

### General Procedure 2 (Conditions B)

To a stirred solution of amine (1 mmol) in CH_3_CN (3 mL) H_2_O_2_ 30% v/v (2 mmol, 227 μL) was added. The resulting solution was stirred at room temperature for the time indicated in Table 2, and CH_2_Cl_2_ (10 mL) and water (10 mL) were added. The organic layer was separated, and the aqueous phase was extracted with dichloromethane (15 mL). The combined organic layers were washed with brine, dried over MgSO_4_, and evaporated under reduced pressure. The crude mixture was purified by flash column chromatography over deactivated silica gel to afford the corresponding nitrone.

### *N*-4-Fluorobenzylideneisopropylamine *N*-Oxide (2k)

Following the general procedure A, the reaction of *N*-(4-fluorobenzyl)-2-propanamine (**1k**) (217 mg, 1.30 mmol) with H_2_O_2_ (5,2 mmol, 554 μL) in MeOH (4 mL) at 50 °C (oil bath) afforded after purification by silica gel flash chromatography (EtOAc) the nitrone **2k** (193 mg, 82%, yellow oil). Following the general procedure B, the reaction of *N*-(4-fluorobenzyl)-2-propanamine (**1k**) (250 mg, 1.50 mmol) with H_2_O_2_ (3 mmol, 340 mL) in CH_3_CN (4 mL) at rt, afforded after purification by silica gel flash chromatography (EtOAc) the nitrone **2k** (241 mg, 89%, yellow oil). ^1^H NMR (300 MHz, CDCl_3_): δ 8,34–8.32 (m, 2H), 7.49 (s, 1H), 7.25–7.23 (m, 2H), 4.24 (sep, *J* = 7.1 Hz, 1H), 1.57 (d, *J* = 7.1 Hz, 6H). ^13^C{^1^H} NMR (75 MHz, CDCl_3_): δ 164.8, 161.5, 130.7, 130.6, 115.6, 115.4, 67.7, 20.9. HRMS (TOF MS EI+): calculated for C_10_H_12_NOF, 181.0903; found, 181.0902 ([M^+^], 56).

### 6-Dimethylamino-3,4-Dihydroisoquinoline *N*-Oxide (2o)

Following the general procedure A, the reaction of 6-(dimethylamino)-1,2,3,4-tetrahydroisoquinoline (174 mg, 1 mmol) with H_2_O_2_ (4 mmol, 453 μL) in MeOH (4 mL) at 50 °C (oil bath) afforded after purification by silica gel flash chromatography (CH_2_Cl_2_/MeOH 9/1) the nitrone **2o** (150 mg, 79%, yellow oil). ^1^H NMR (300 MHz, CDCl_3_): δ 7,68 (s, 1H), 7.12–7.08 (m, 1H), 6.57–6.54 (m, 2H), 4.12 (t, *J* = 7.1 Hz, 2H), 3.15 (t, *J* = 7.1 Hz, 2H), 3.01 (s, 6H). ^13^C{^**1**^H}NMR (75 MHz, CDCl): δ 151.1, 135.5, 131.5, 127.3, 116.0, 110.6, 57.2, 39.8, 28.2. HRMS (ESI+): Calculated for C_11_H_15_N_2_O, 191.1181; found, 191.1179 ([M + H], 100).

### Typical Procedure for the Cycloaddition Reaction. Cycloaduct (7)

To a stirred solution of tetrahydroisoquinoline **1a** (0.5 mmol, 66 mg) in MeOH (2 mL) H_2_O_2_ 30% v/v (2 mmol, 277 μL) was added. The resulting solution was stirred at 50 °C (oil bath) for 12 h and *N*-methyl maleimide (0.5 mmol, 56 mg) was added. The reaction was stirred at 50 °C (oil bath) for 4 h, and CH_2_Cl_2_ (10 mL) and water (10 mL) were added, the organic layer was separated, and the aqueous phase was extracted with dichloromethane (10 mL). The combined organic layers were washed with brine, dried over MgSO_4_, and evaporated under reduced pressure. The crude mixture was purified by flash column chromatography (CH_2_Cl_2_/MeOH 99/1) to afford nitrone **7** (97 mg, 75%, yellow oil). Spectroscopic data match those previously reported.^33^

## Supporting Information Available

^1^H and ^13^C NMR and ESI/MS spectra for all compounds and solvent specifications (PDF)

## References

[ref1] aRevuelta J.; Cicchi S.; Goti A.; Brandi A. Enantiopure Cyclic Nitrones: A Useful Class of Building Blocks for Asymmetric Syntheses. Synthesis 2007, 2007, 485 10.1055/s-2007-965914.

[ref2] aXiong J.; Meng W.-J.; Zhang H.-Y.; Zou Y.; Wang W.-X.; Wang X.-Y.; Yang Q.-L.; Osman E. E. A.; Hu J.-F. Lycofargesiines A–F, Further *Lycopodium* Alkaloids from the Club Moss. Phytochemistry 2019, 162, 183 10.1016/j.phytochem.2019.03.015. 30928888

[ref3] aZhang Z.; Zhang G.; Sun Y.; Szeto S. S. W.; Law H. C. H.; Quan Q.; Li G.; Yu P.; Sho E.; Siu M. K. W.; Lee S. M. Y.; Chu I. K.; Wang Y. Tetramethylpyrazine Nitrone, a Multifunctional Neuroprotective Agent for Ischemic Stroke Therapy. Sci. Rep. 2016, 6, 37148 10.1038/srep37148. 27841332 PMC5107909

[ref4] aYang M.; Liang X.; Zhang Y.; Ouyang Z.; Dong W. A Nitronyl Nitroxide and its Two 1D Chain Cu–Tb Complexes: Synthesis, Structures, and Magnetic Properties. RSC Adv. 2020, 10, 8490 10.1039/D0RA00018C. PMC9050009 35497860

[ref5] aDeletraz A.; Zéamari K.; Hua K.; Combes M.; Villamena F. A.; Tuccio B.; Callizot N.; Durand G. Substituted α-Phenyl and α-Naphthlyl-*N*-*tert*-butyl Nitrones: Synthesis, Spin-Trapping, and Neuroprotection Evaluation. J. Org. Chem. 2020, 85, 6073 10.1021/acs.joc.0c00563. 32267700

[ref6] aMerino P. New Developments in Nucleophilic Additions to Nitrones. C. R. Chim. 2005, 8, 775 10.1016/j.crci.2005.02.013.

[ref7] aSynthetic Applications of 1,3-Dipolar Cycloaddition Chemistry toward Heterocycles and Natural Products. Padwa A., Pearson W. H., Eds.; Wiley: New York, Chichester, 2002.

[ref8] aMerino P. Nitrones and Cyclic Analogues. An Update. Science of Synthesis; Thieme: Stuttgart, 2010; Vol. 4, pp 325.

[ref9] aSalehzadeh H.; Mashhadizadeh M. H. Nitrone Synthesis via Pair Electrochemical Coupling of Nitro-compounds with Benzyl alcohol Derivatives. J. Org. Chem. 2019, 84, 9307 10.1021/acs.joc.9b00736. 31194555

[ref10] aMatassini C.; Parmeggiani C.; Cardona F.; Goti A. Oxidation of *N*,*N*-Disubstituted Hydroxylamines to Nitrones with Hypervalent Iodine Reagents. Org. Lett. 2015, 17, 4082 10.1021/acs.orglett.5b02029. 26225452

[ref11] aSoldaini G.; Cardona F.; Goti A. Catalytic Oxidation of Imines Based on Methyltrioxorhenium/Urea Hydrogen Peroxide: A Mild and Easy Chemo-and Regioselective Entry to Nitrones. Org. Lett. 2007, 9, 473 10.1021/ol062862w. 17249790

[ref12] aKalhor M.; Samiei S.; Mirshokraei S. A. Facile One-pot Synthesis of Novel N-benzimidazolyl-α-arylnitrones Catalyzed by Salts of Transition Metals. RSC Adv. 2019, 9, 41851 10.1039/C9RA08570J. PMC9076553 35541607

[ref13] Morozov D. A.; Kirilyuk I. A.; Komarov D. A.; Goti A.; Bagryanskaya I. Y.; Kuratieva N. V.; Grigorev I. A. Synthesis of a Chiral *C*_2_-Symmetric Sterically Hindered Pyrrolidine Nitroxide Radical via Combined Iterative Nucleophilic Additions and Intramolecular 1,3-Dipolar Cycloadditions to Cyclic Nitrones. J. Org. Chem. 2012, 77, 10688 10.1021/jo3019158. 23130653

[ref14] Murahashi S.-I.; Ohtake H.; Imada Y. Synthesis of (*R*)- and (*S*)-3-(tert-Butyldimethylsilyloxy)-1-pyrroline N-Oxides-chiral Nitrones for Synthesis of Biologically active Pyrrolidine Derivative, Geissman-Waiss Lactone. Tetrahedron Lett. 1998, 39, 2765 10.1016/S0040-4039(98)00333-5.

[ref15] aBaran P. S.; Hafensteiner B. D.; Ambhaikar N. B.; Guerrero C. A.; Gallagher J. D. Enantioselective Total Synthesis of Avrainvillamide and the Stephacidins. J. Am. Chem. Soc. 2006, 128, 8678 10.1021/ja061660s. 16802835

[ref16] Mitsui H.; Zenki S.-i.; Shiota T.; Murahashi S.-I. Tungstate Catalysed Oxidation of Secondary Amines with Hydrogen Peroxide. A Novel Transformation of Secondary Amines into Nitrones. J. Chem. Soc., Chem. Commun. 1984, 874 10.1039/c39840000874.

[ref17] Murahashi S.-I.; Shiota T. Selenium dioxide Catalyzed Oxidation of Secondary Amines with Hydrogen peroxide. Simple Synthesis of Nitrones from Secondary Amines. Tetrahedron Lett. 1987, 28, 2383 10.1016/S0040-4039(00)96130-6.

[ref18] aGoti A.; Cardona F.; Soldaini G. A Large-scale Low-cost Preparation of N-benzylhydroxylamine hydrochloride. Org. Synth. 2005, 81, 204 10.1002/0471264229.os081.22.

[ref19] aZonta C.; Cazzola E.; Mba M.; Licini G. C3-Symmetric Titanium(IV) Triphenolate Amino Complexes for a Fast and Effective Oxidation of Secondary Amines to Nitrones with Hydrogen Peroxide. Adv. Synth. Catal. 2008, 350, 2503 10.1002/adsc.200800494.

[ref20] Colladon M.; Scarso A.; Strukul G. Mild Catalytic Oxidation of Secondary and Tertiary Amines to Nitrones and N-oxides with H_2_O_2_ mediated by Pt(II) catalysts. Green Chem. 2008, 10, 793 10.1039/b805404e.

[ref21] aAbrantes M.; Gonçalves I. S.; Pillinger M.; Vurchio C.; Cordero F. M.; Brandi A. Molybdenum Oxide/bipyridine Hybrid Material {[MoO_3_(bipy)][MoO_3_(H_2_O)]} as Catalyst for the Oxidation of Secondary Amines to Nitrones. Tetrahedron Lett. 2011, 52, 7079 10.1016/j.tetlet.2011.10.079.

[ref22] Forcato M.; Mba M.; Nugent W. A.; Licini G. Effective Oxidation of Secondary Amines to Nitrones with Alkyl Hydroperoxides Catalysed by (Trialkanolaminato)titanium(IV) Complexes. Eur. J. Org. Chem. 2010, 2010, 740 10.1002/ejoc.200900867.

[ref23] Gella C.; Ferrer E.; Alibes R.; Busque F.; de March P.; Figueredo M.; Font J. A Metal-free General Procedure for Oxidation of Secondary Amines to Nitrones. J. Org. Chem. 2009, 74, 6365 10.1021/jo901108u. 19606814

[ref24] Murray R. W.; Singh M. Chemistry of dioxiranes. 16. A Facile One-step Synthesis of C-aryl Nitrones Using Dimethyldioxirane. J. Org. Chem. 1990, 55, 2954 10.1021/jo00296a073.

[ref25] Looper R. E.; Williams R. M. A Concise Asymmetric Synthesis of the Marine Hepatotoxin 7-Epicylindrospermopsin. Angew. Chem., Int. Ed. 2004, 43, 2930 10.1002/anie.200454208. 15170307

[ref26] Zajac W. W. Jr.; Walters T. R.; Darcy M. G. Oxidation of Amines with 2-Sulfonyloxaziridines (Davis’ reagents). J. Org. Chem. 1988, 53, 5856 10.1021/jo00260a012.

[ref27] Iida H.; Imada Y.; Murahashi S.-I. Biomimetic Flavin-catalysed Reactions for Organic Synthesis. Org. Biomol. Chem. 2015, 13, 7599 10.1039/C5OB00854A. 26077635

[ref28] A minor amount of regioisomeric nitrone (less than 10%) was observed in the ^1^H NMR spectrum of the crude reaction mixture.

[ref29] aCooper M. S.; Heaney H.; Newbold A. J.; Sanderson W. R. Oxidation Reactions Using Urea-Hydrogen Peroxide; A Safe Alternative to Anhydrous Hydrogen Peroxide. Synlett 1990, 1990, 533 10.1055/s-1990-21156.

[ref30] aRusso A.; Lattanzi A. Hydrogen-Bonding Catalysis: Mild and Highly Chemoselective Oxidation of Sulfides. Adv. Synth. Catal. 2009, 351, 521 10.1002/adsc.200900020.

[ref31] aRavikumar K. S.; Bégué J.-P.; Bonnet-Delpon D. A Selective Conversion of Sulfide to Sulfoxide in Hexafluoro-2-propanol. Tetrahedron Lett. 1998, 39, 3141 10.1016/S0040-4039(98)00498-5.

[ref32] aPayne G. B.; Deming P. H.; Williams P. H. Reactions of Hydrogen Peroxide. VII. Alkali-Catalyzed Epoxidation and Oxidation Using a Nitrile as Co-reactant. J. Org. Chem. 1961, 26, 659 10.1021/jo01062a004.

[ref33] aFurnival R. C.; Saruengkhanphasit R.; Holberry H. E.; Shewring J. R.; Guerrand H. D. S.; Adams H.; Coldham I. Cascade Oxime Formation, Cyclization to a Nitrone, and Intermolecular Dipolar Cycloaddition. Org. Biomol. Chem. 2016, 14, 10953 10.1039/C6OB01871H. 27819376

[ref34] Clementson S.; Radaelli A.; Fjelbye K.; Tanner D.; Jessing M. Strain-Release Driven Cycloadditions for Rapid Construction of Functionalized Pyridines and Amino Alcohols. Org. Lett. 2019, 21, 4763 10.1021/acs.orglett.9b01652. 31180685

[ref35] Brandi A.; Garro S.; Guarna A.; Goti A.; Cordero F.; De Sarlo F. Rearrangement of isoxazoline-5-spiro derivatives. 2. Synthesis and rearrangement of tetrahydroisoxazole-5-spirocyclopropanes. Preparation of precursors of quinolizine, isoquinoline, and indole alkaloids. J. Org. Chem. 1988, 53, 2430 10.1021/jo00246a008.

[ref36] aSchmitz E. Isochinolin, II. 3.4-Dihydro-isochinolin-*N*-oxyd. Chem. Ber. 1958, 91, 1488 10.1002/cber.19580910719.

[ref37] Ogata Y.; Sawaki Y. Peracid oxidation of imines. Kinetics and mechanism of competitive formation of nitrones and oxaziranes from cyclic and acyclic imines. J. Am. Chem. Soc. 1973, 95, 4692 10.1021/ja00795a037.

[ref38] Cherest M.; Lusinchi X. Reaction des nitrones avec les chlorures d’acides: action de chlorures d’aryl-sulfonyles et du chlorure de benzoyle sur des n-oxy (aryl-1 dihydro-3,4 isoquinoleines). Formation d’une isoquinoleine, d’un isocarbostyryle ou d’une indoline selon les conditions. Tetrahedron 1982, 38, 3471 10.1016/0040-4020(82)85031-X.

[ref39] Jost S.; Gimbert Y.; Greene A. E.; Fotiadu F. Totally Stereocontrolled Nitrone–Ketene Acetal Based Synthesis of (2*S*,3*S*)-N-Benzoyl- and N-Boc-phenylisoserine. J. Org. Chem. 1997, 62, 6672 10.1021/jo971009v.

[ref40] Andrade M. M.; Barros M. T.; Pinto R. C. Exploiting microwave-assisted neat procedures: synthesis of *N*-aryl and *N*-alkylnitrones and their cycloaddition en route for isoxazolidines. Tetrahedron 2008, 64, 10521 10.1016/j.tet.2008.08.101.

[ref41] Colonna S.; Pironti V.; Carrea G.; Pasta P.; Zambianchi F. Oxidation of secondary amines by molecular oxygen and cyclohexanone monooxygenase. Tetrahedron 2004, 60, 569 10.1016/j.tet.2003.10.100.

[ref42] Delso I.; Melicchio A.; Isasi A.; Tejero T.; Merino P. Evasive Neutral 2-Aza-Cope Rearrangements. Kinetic and Computational Studies with Cyclic Nitrones. Eur. J. Org. Chem. 2013, 2013, 5721 10.1002/ejoc.201300836.

[ref43] Zeng Y.; Smith B. T.; Hershberger J.; Aubé J. Rearrangements of Bicyclic Nitrones to Lactams: Comparison of Photochemical and Modified Barton Conditions. J. Org. Chem. 2003, 68, 8065 10.1021/jo035004b. 14535783

