# Supplementary material for: Metal-Free Solvent Promoted Oxidation of Benzylic Secondary Amines to Nitrones with H2O2
Source: J Org Chem. 2021 Sep 16;86(19):13817–23. doi: 10.1021/acs.joc.1c01888 (PMC8650016; doi:10.1021/acs.joc.1c01888)
Supplement: Supplementary file 1 — jo1c01888_si_001.pdf [file jo1c01888_si_001.pdf]

Supporting information for

Metal Free Solvent Promoted Oxidation of Benzylic Secondary Amines to Nitrones with H_2O_2

Álisson Silva Granato,[a,c] Giovanni Wilson Amarante,[c] and Javier Adrio*[a,b]

[a]Departamento de Química Orgánica, Facultad de Ciencias, Universidad Autónoma de Madrid, Cantoblanco, 28049 Madrid, Spain

[b]Institute for Advanced Research in Chemical Sciences (IAdChem), Universidad Autónoma de Madrid, 28049 Madrid, Spain

[c]*Chemistry Department, Federal University of Juiz de Fora, Sao Pedro, Juiz de Fora, 36036-900, Brazil*

Table of content

1. NMR Spectra collection S-2
2. ESI/MS of the oxidation of tetraisoquinoline **1a** S-11
3. Solvent Specifications……………………………………………………………S12

NMR Spectra collection

^{1}H NMR (300 MHz, $CDCl_3$)

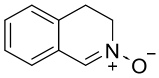

2a

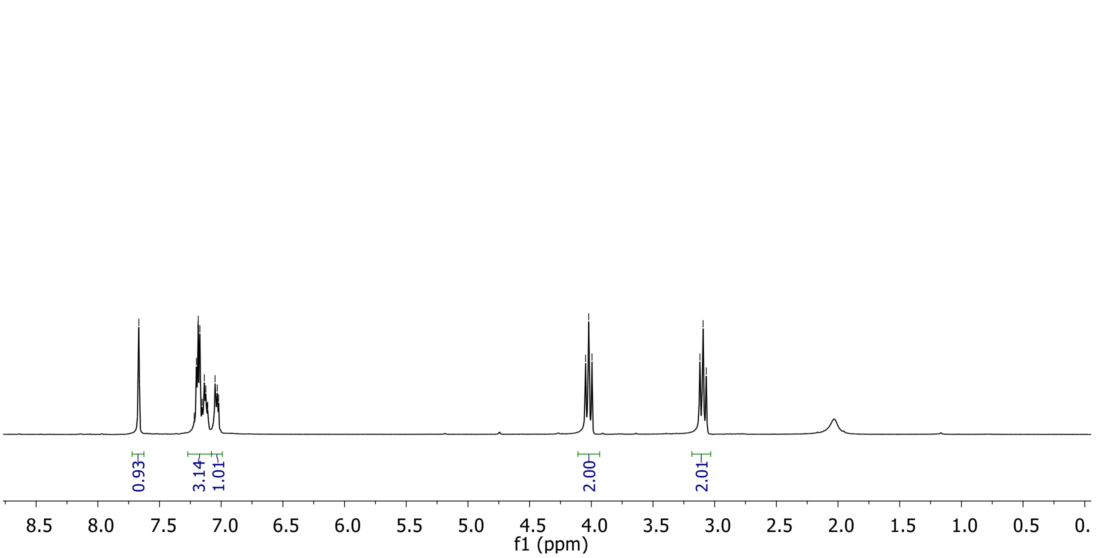

^{1}H NMR (300 MHz, $CDCl_3$)

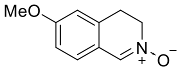

2b

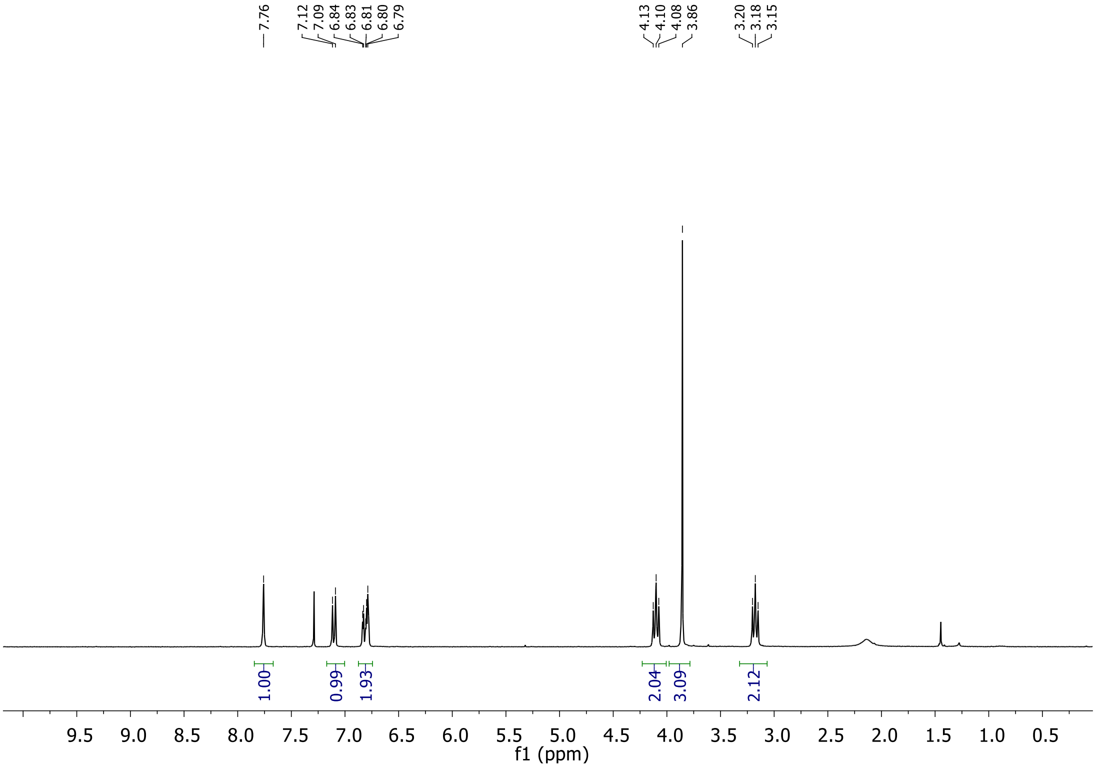

^{1}H NMR (300 MHz, $CDCl_3$)

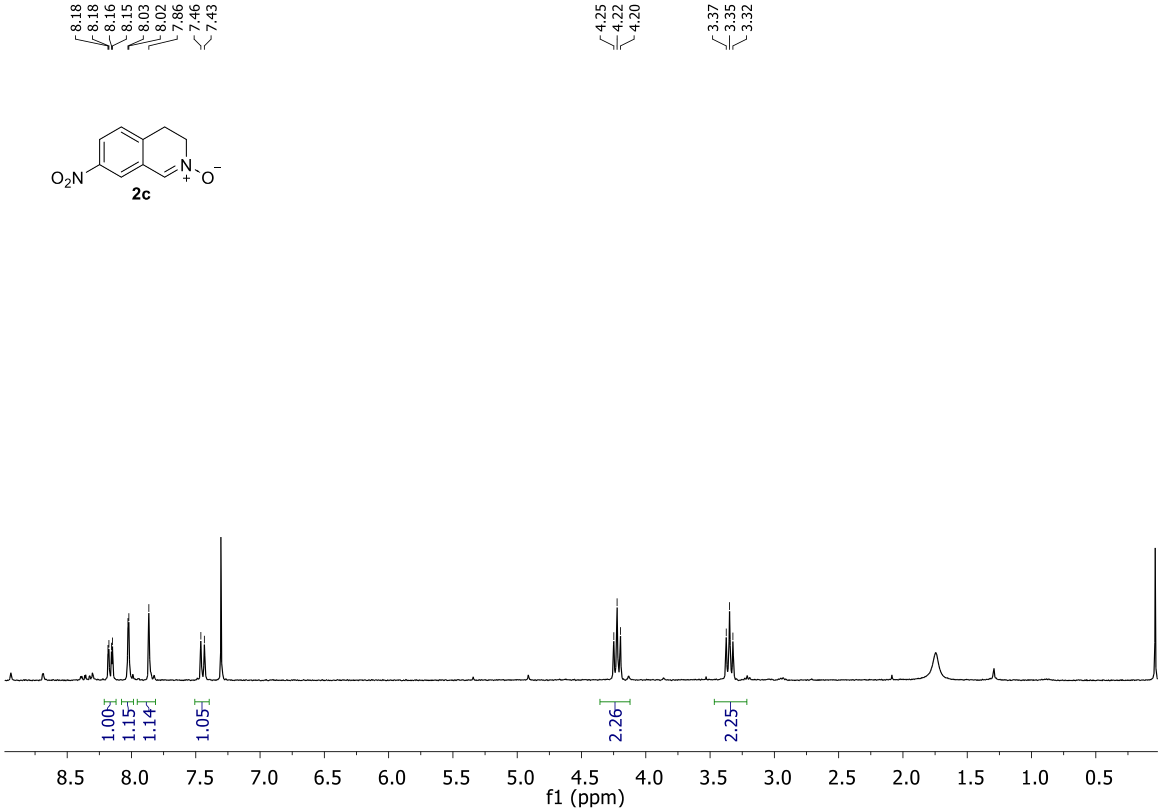

^{1}H NMR (300 MHz, $CDCl_3$)

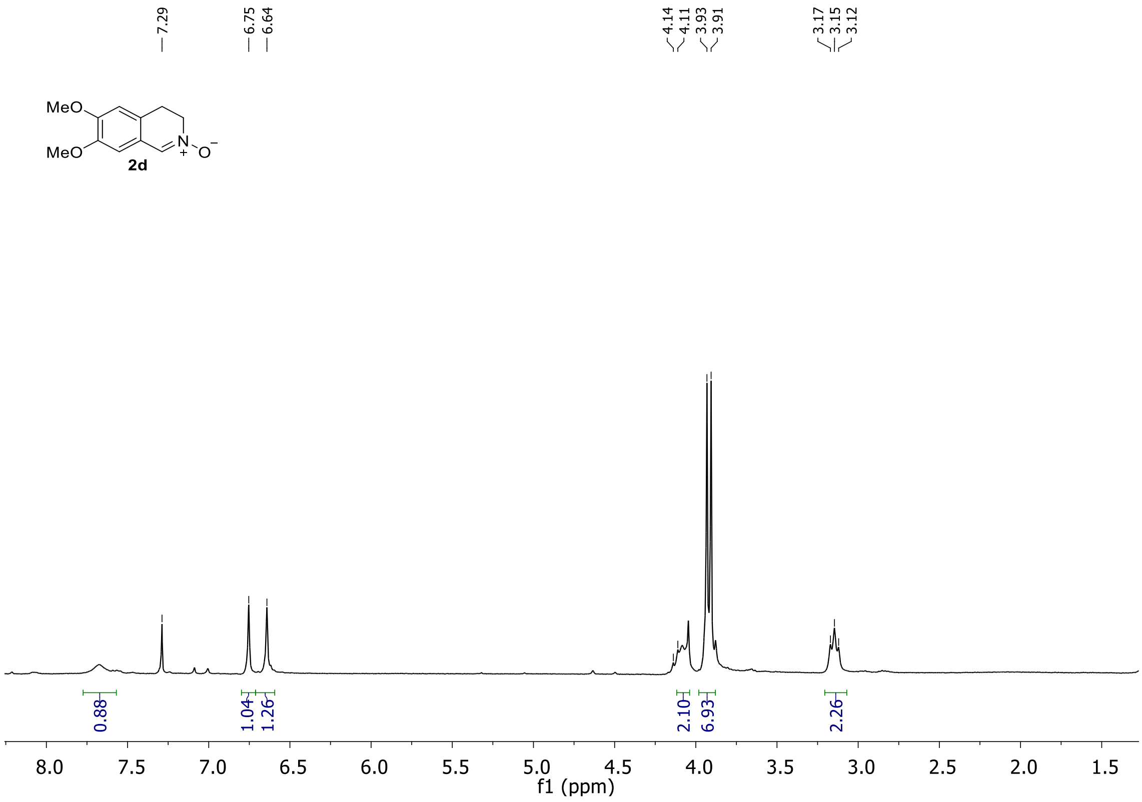

^{1}H NMR (300 MHz, $CDCl_3$)

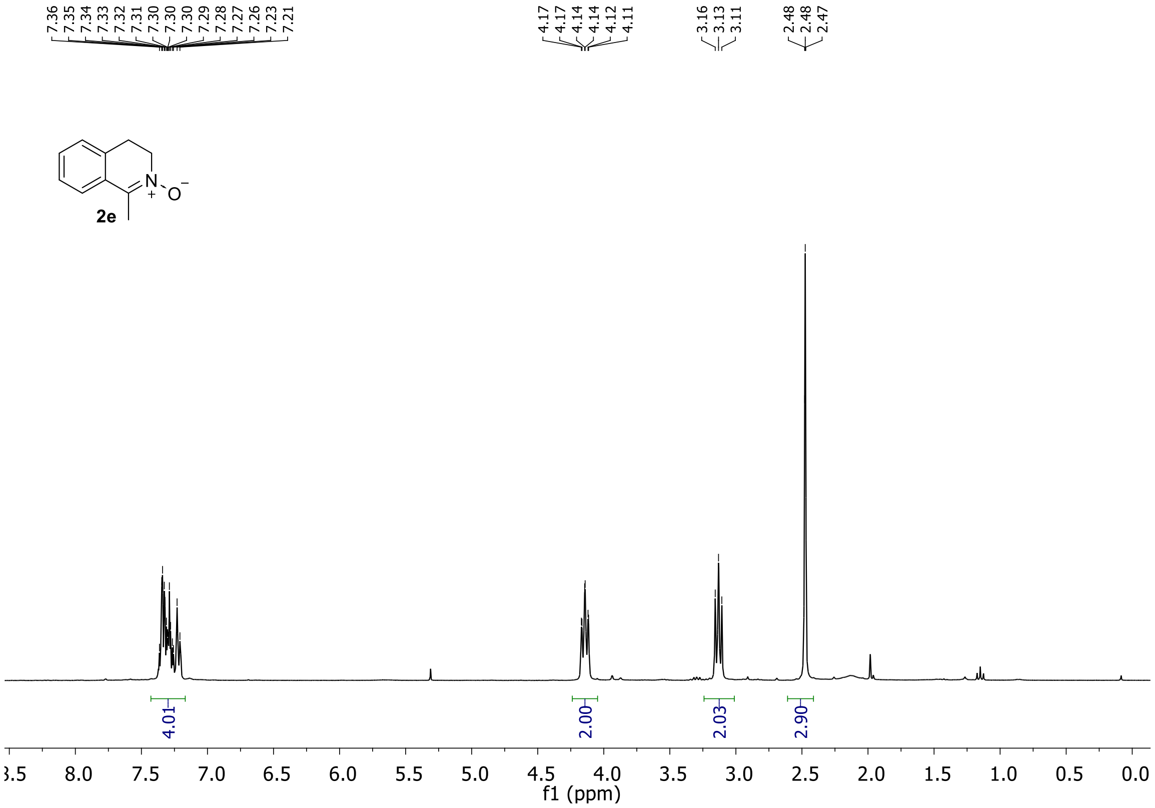

^{1}H NMR (300 MHz, $CDCl_3$)

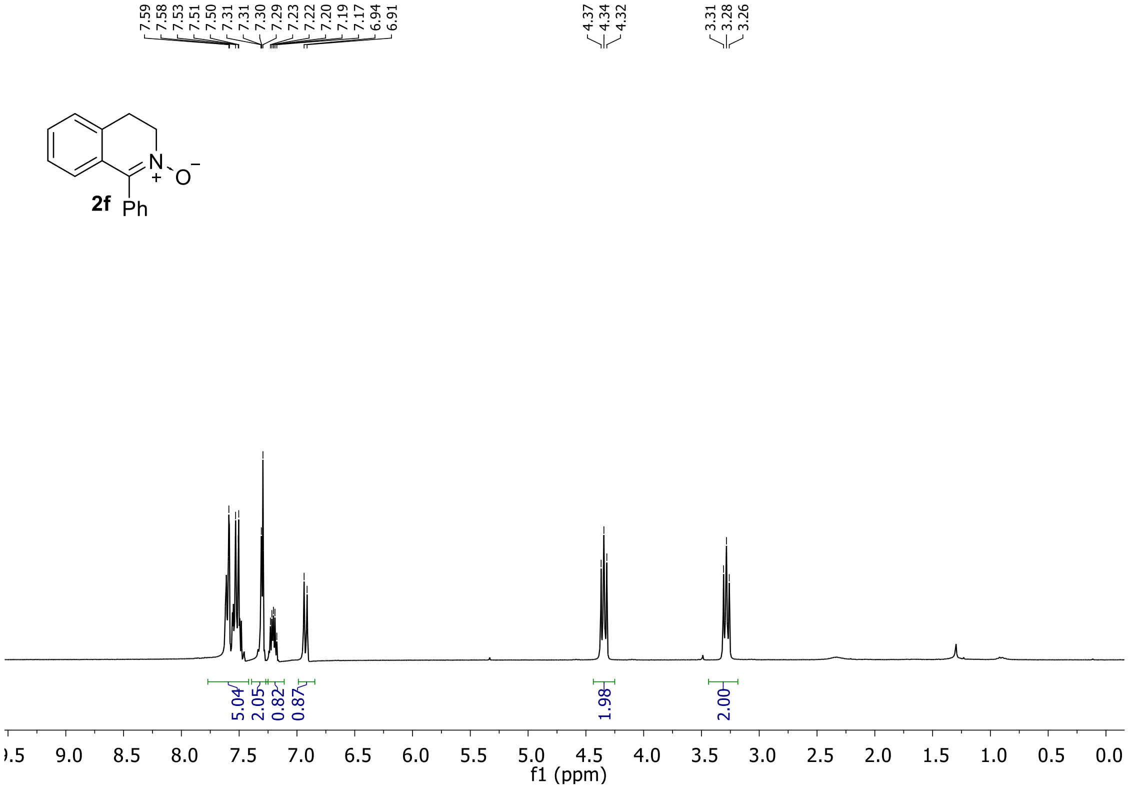

^{1}H NMR (300 MHz, CDCl$_3$)

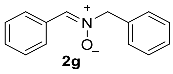

2g

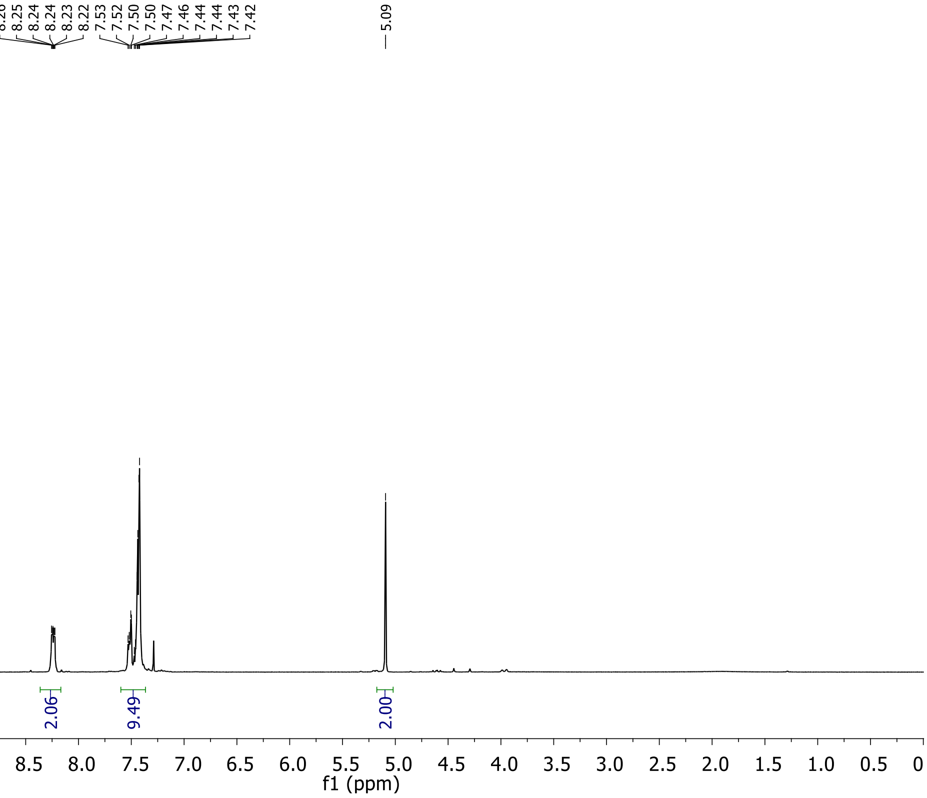

^{1}H NMR (300 MHz, CDCl$_3$)

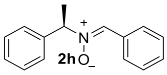

2h

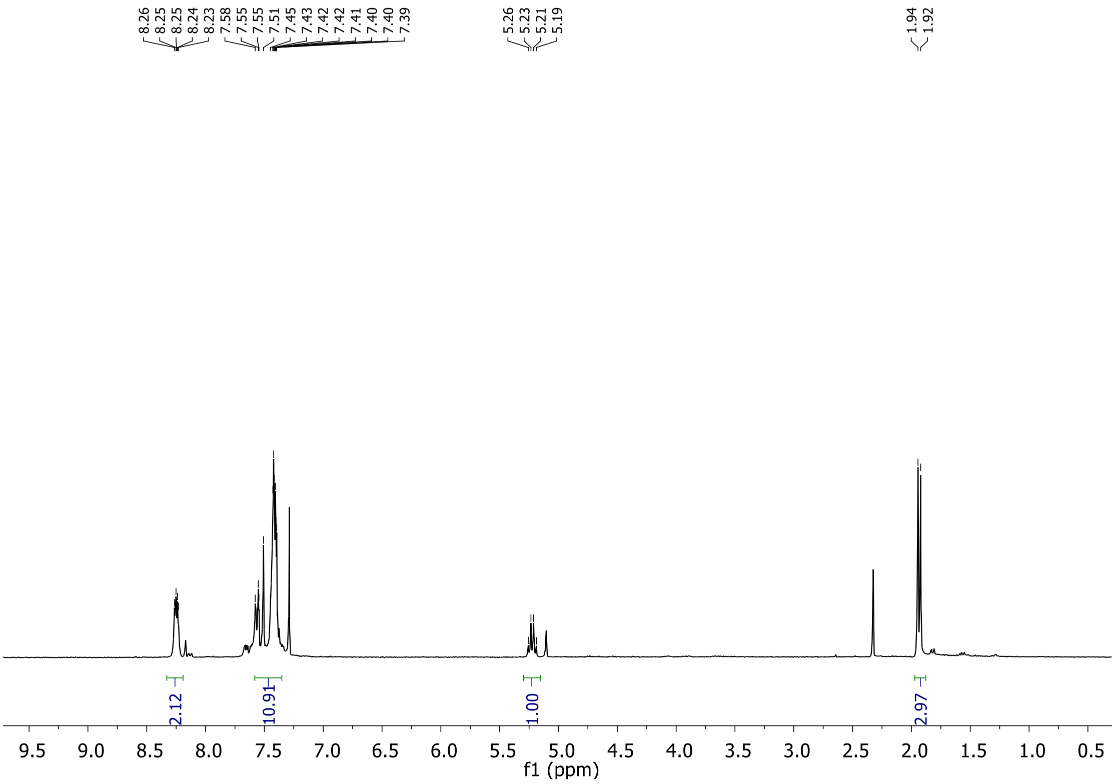

^{1}H NMR (300 MHz, $CDCl_3$)

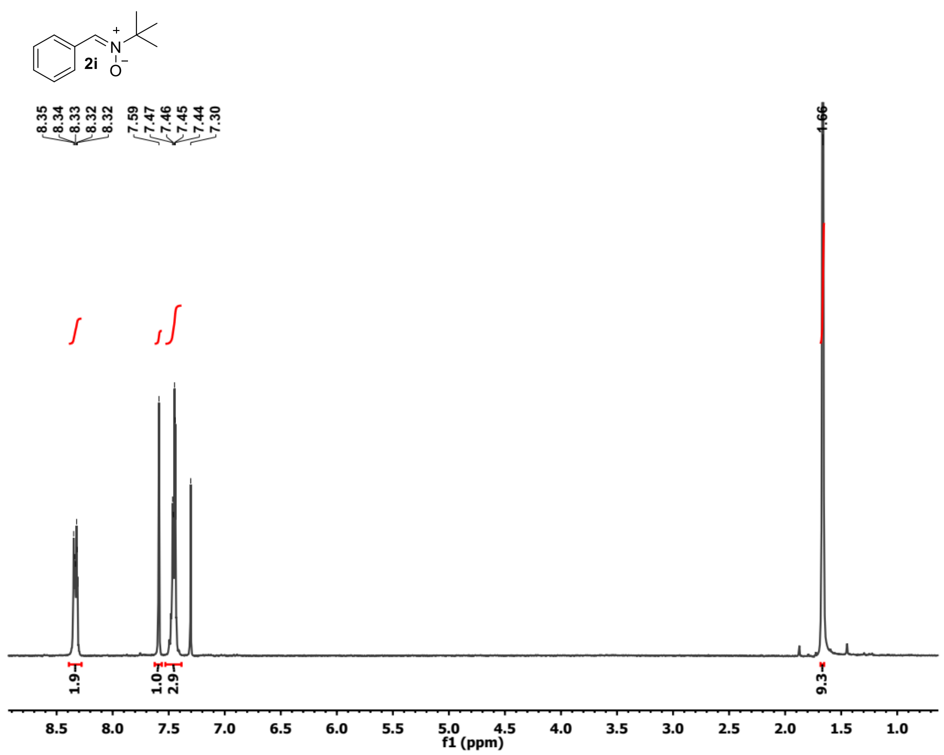

^{1}H NMR (300 MHz, $CDCl_3$)

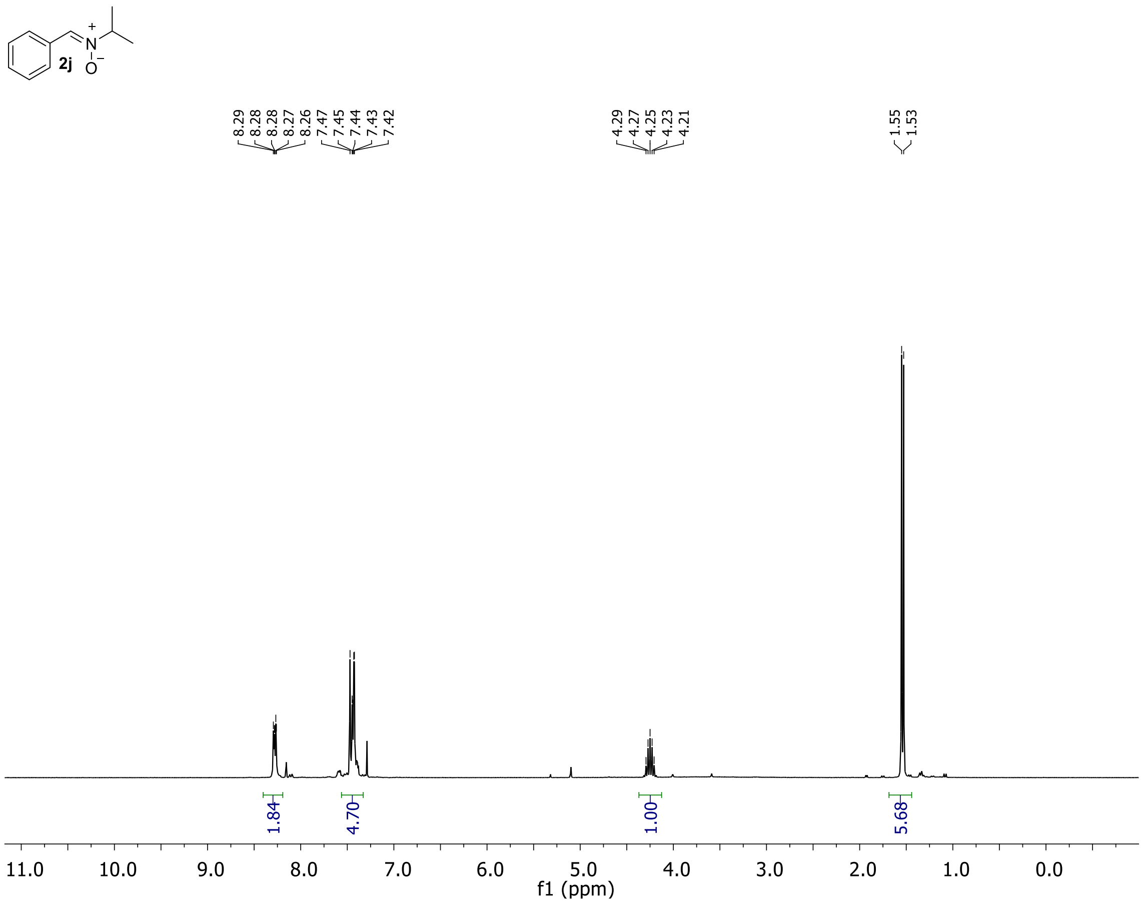

^{1}H NMR (300 MHz, $CDCl_3$)

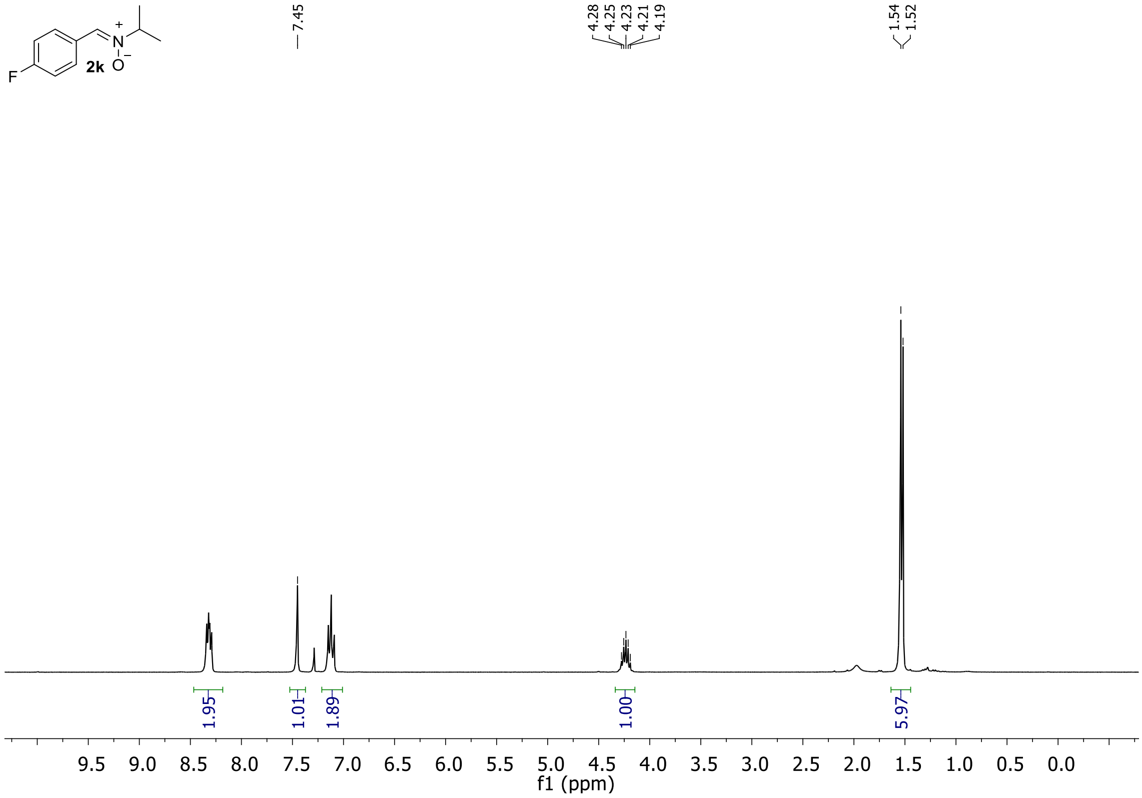

^{13}C{^{1}H}NMR (75 MHz, $CDCl_3$)

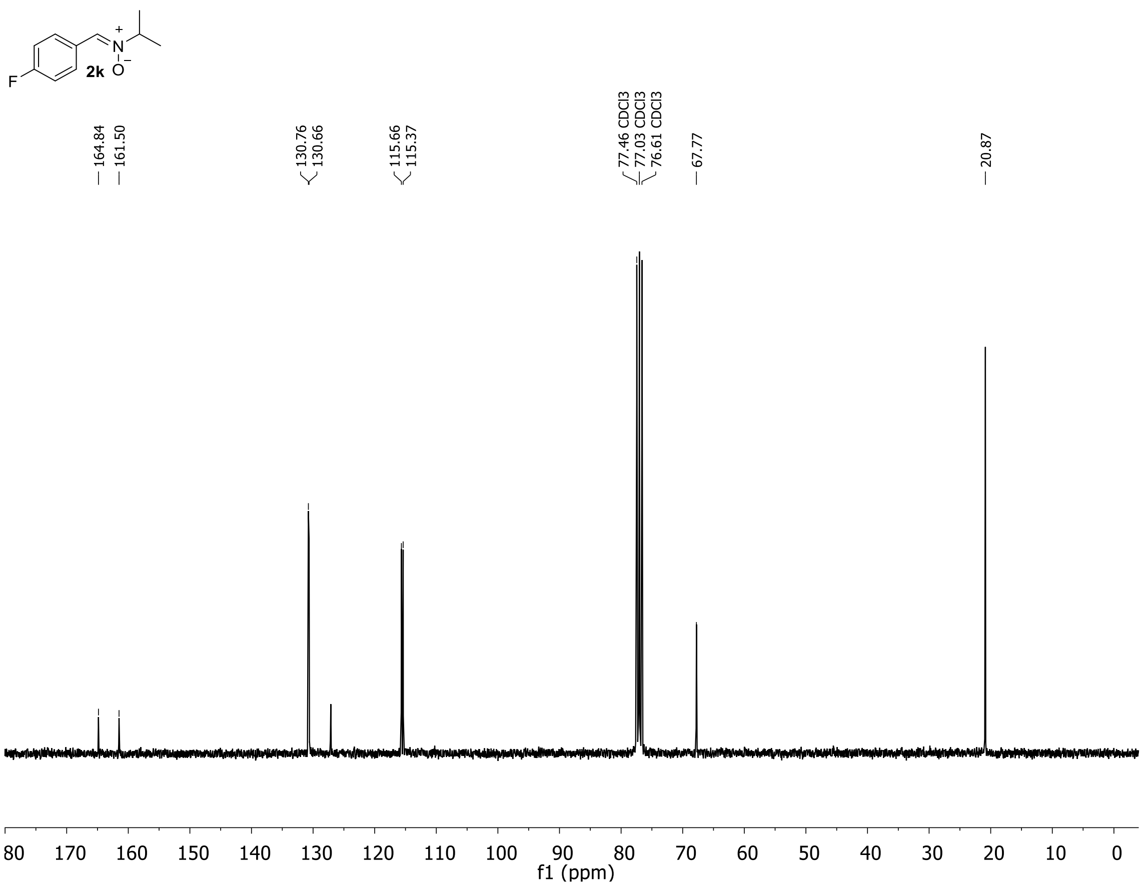

^{1}H NMR (300 MHz, $CDCl_3$)

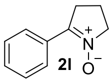

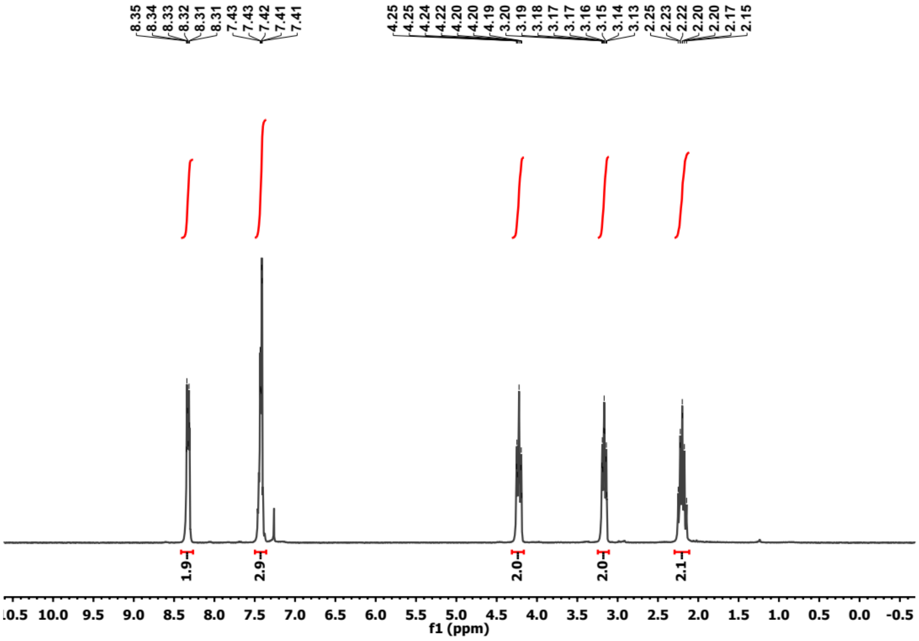

^{1}H NMR (300 MHz, $CDCl_3$)

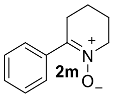

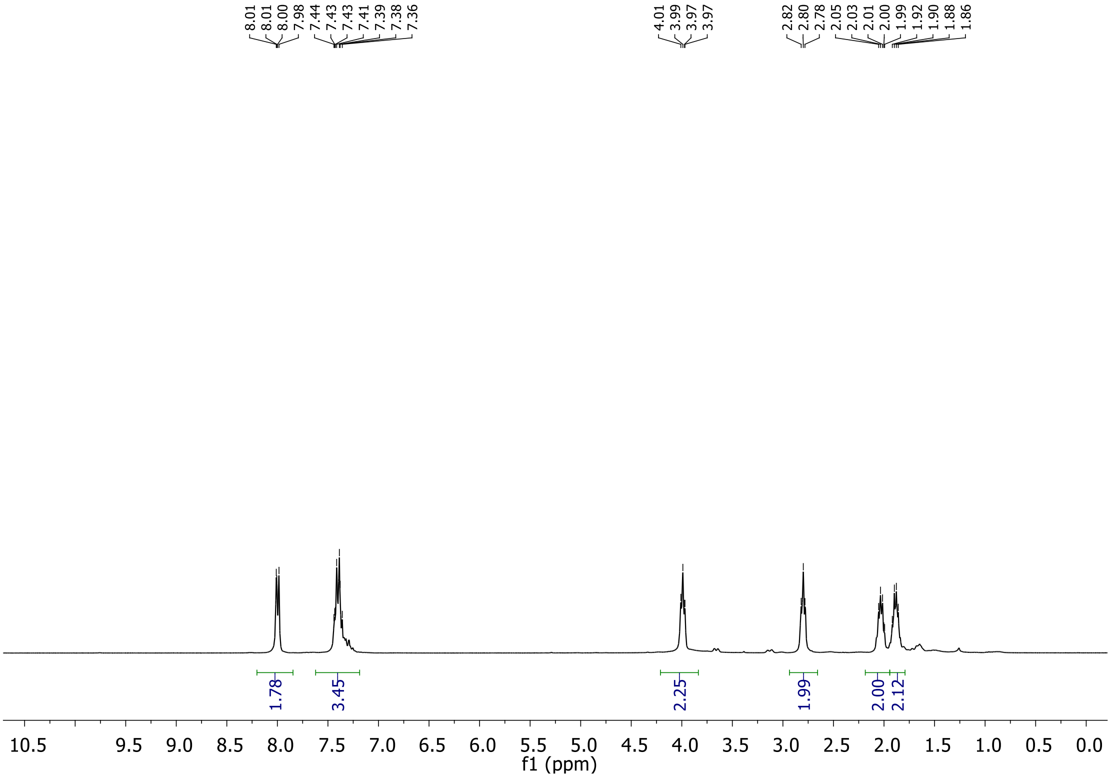

^{1}H NMR (300 MHz, $CDCl_3$)

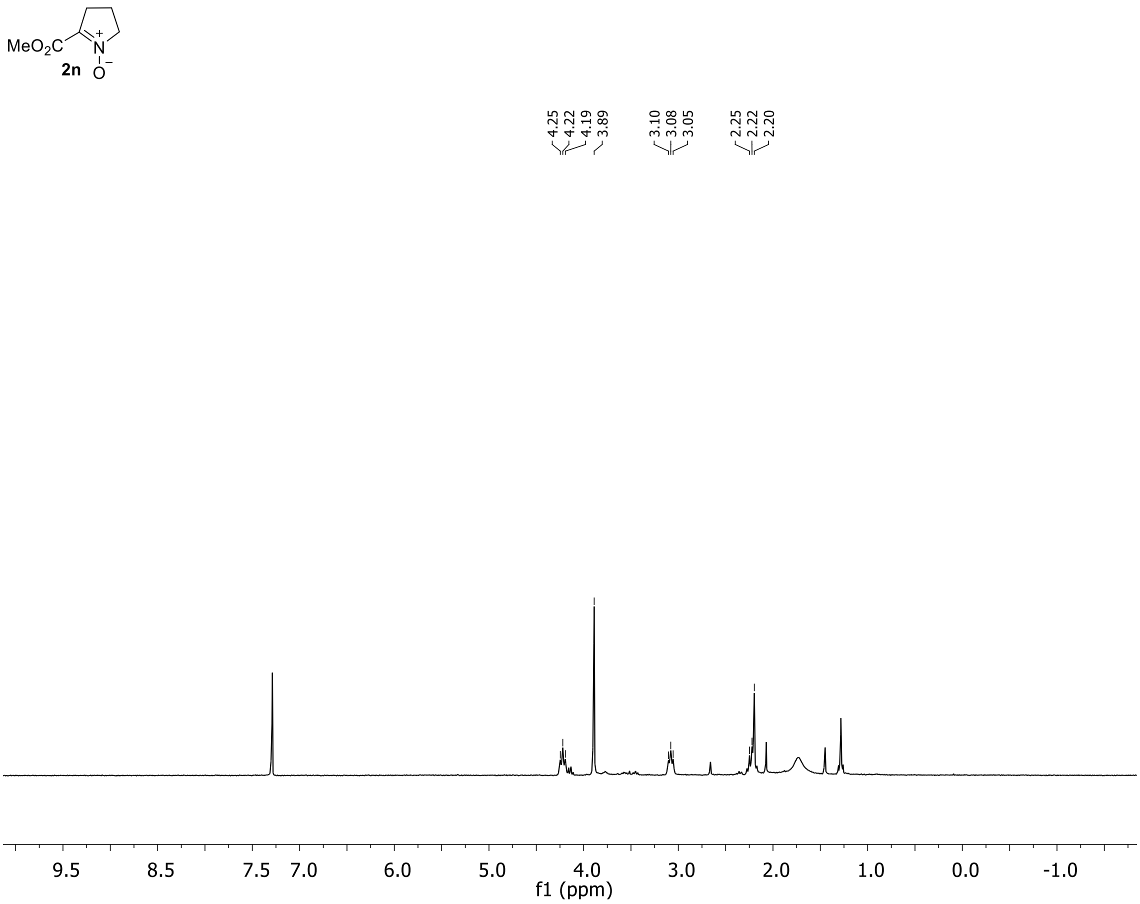

^{1}H NMR (300 MHz, $CDCl_3$)

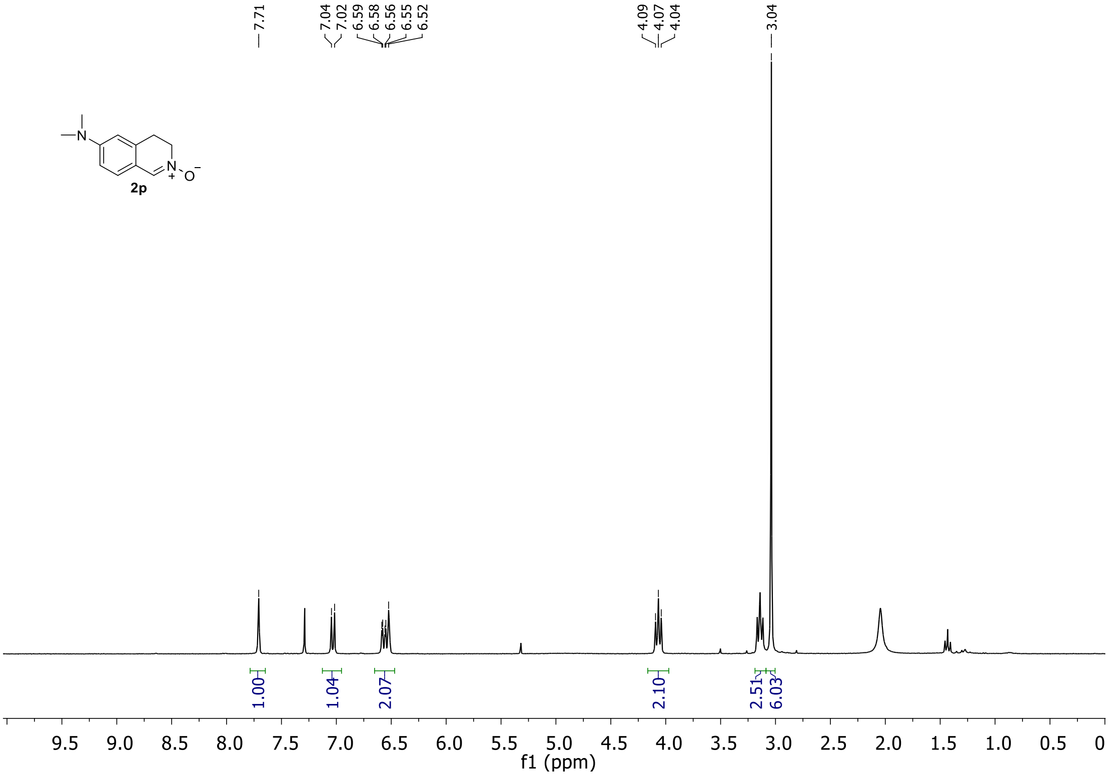

$^{13}C\{^{1}H\}$NMR (75 MHz, $CDCl_3$)

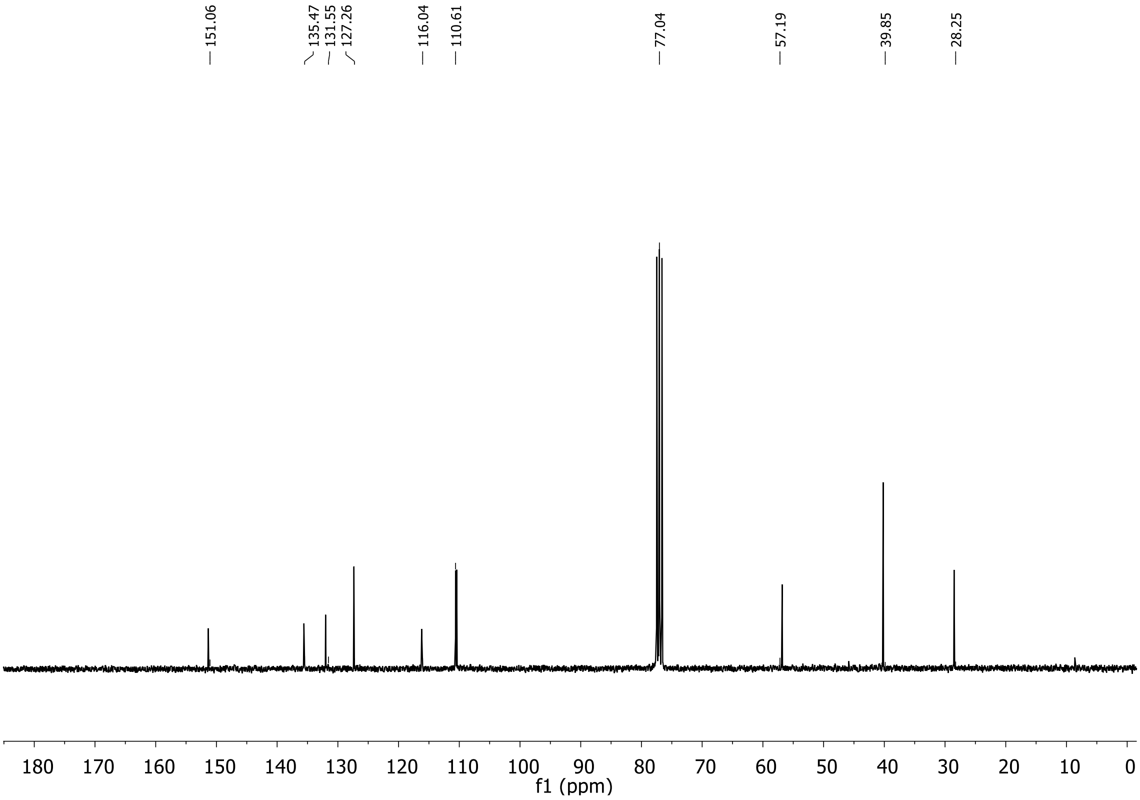

^{1}H NMR (300 MHz, $CDCl_3$)

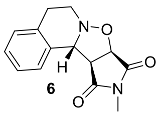

6

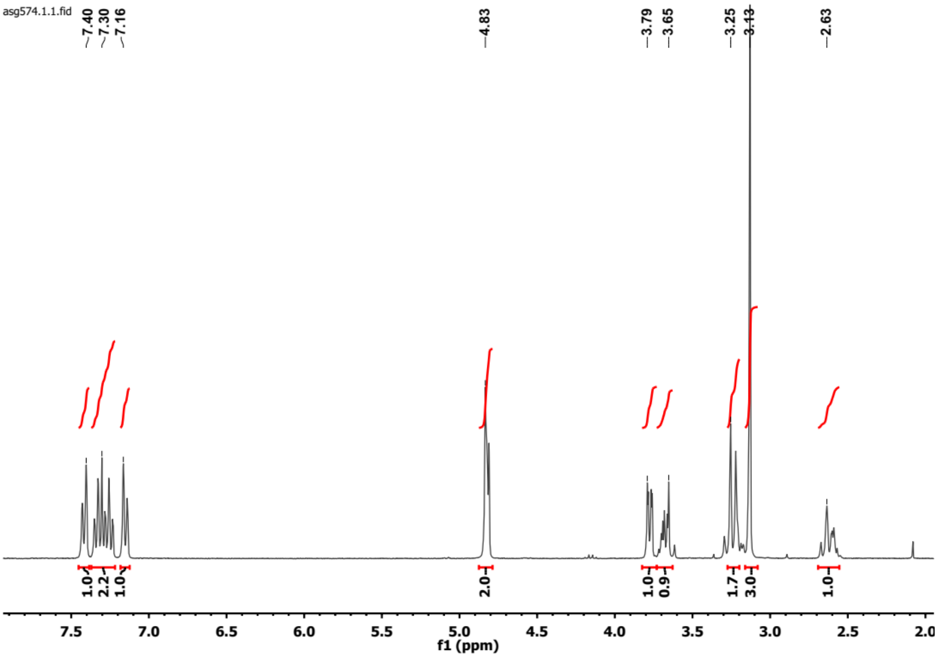

^{1}H NMR (300 MHz, $CDCl_3$)

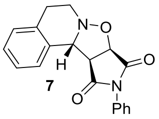

7

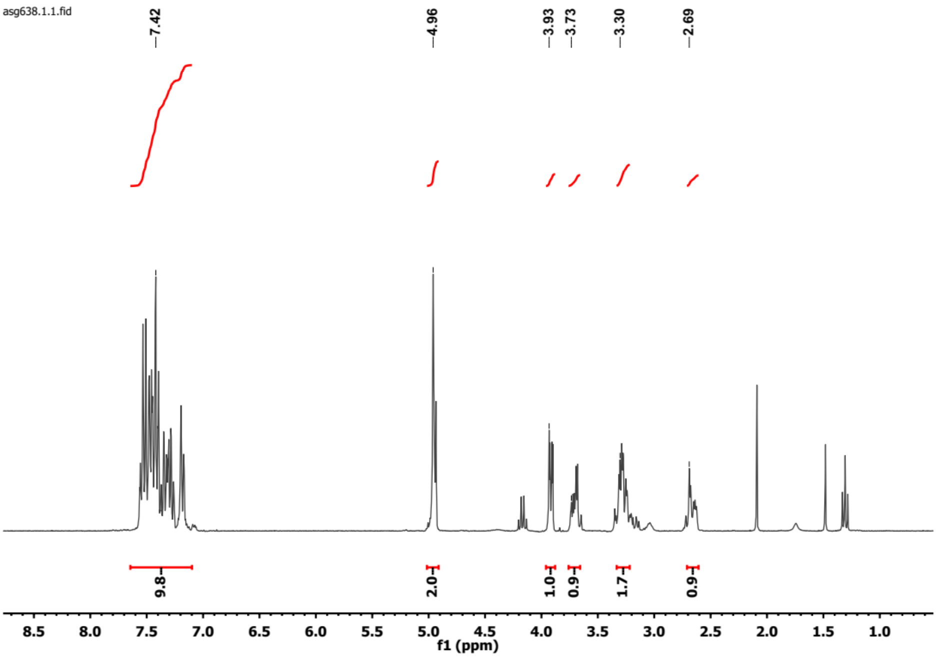

ESI/MS of the reaction of tetraisoquinoline **1a** with H_2O_2 (2 equiv) in CH_3CN as solvent peacks at 60 Da and 80Da corresponding to acetamide (M+H+) and (M+Na+)

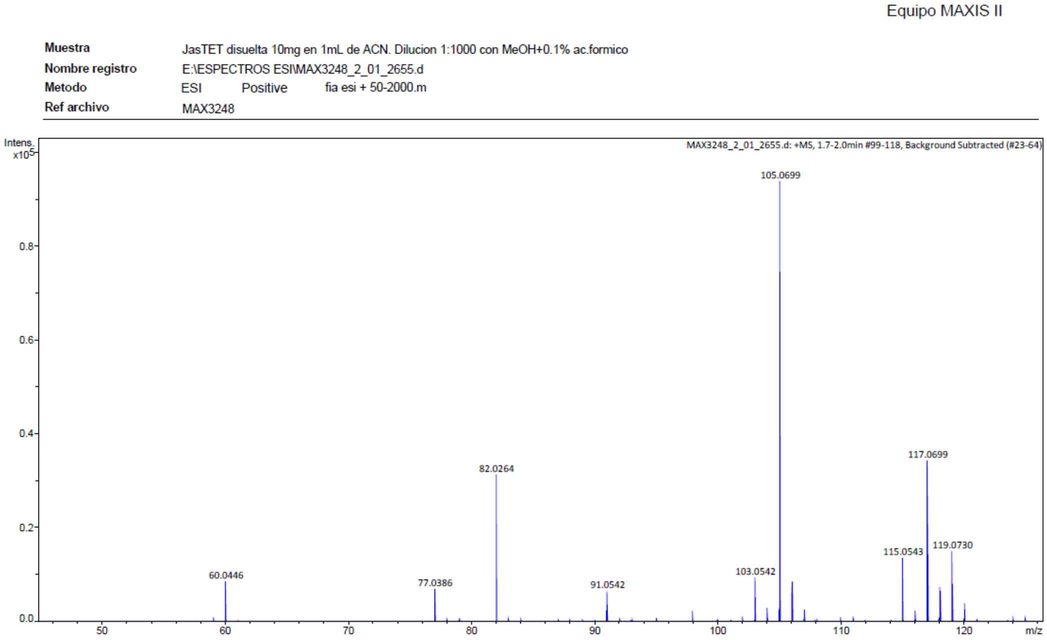

Solvent Specifications

Methanol ≥99.8%, AnalaR NORMAPUR® ACS, Reag. Ph. Eur. analytical reagent (supplier vwr)

Specification Test Results

Appearance	Clear colourless liquid
Assay (on anhydrous substance)	Min. 99.8 %
IR Spectrum	Passes test
Solubility in water	Passes test ACS
Acidity	Max. 0.0002 meq/g
Alkalinity	Max. 0.00006 meq/g
Boiling point	64 - 65 °C
Colouration	Max. 10 APHA
Density (20/4)	0.791 - 0.792
Density (20/20)	0.791 - 0.793
Substances coloured by H_2SO_4	Max. 10 APHA
Acetone + aldehydes (as CH_3COCH_3)	Max. 10 ppm
Acetone	Max. 5 ppm
Acetaldehyde	Max. 5 ppm
Ethanol	Max. 0.1 %
Evaporation residue	Max. 10 ppm
Formaldehyde	Max. 1 ppm
Substances reducing $KMnO_4$ (as O)	Max. 2 ppm
Water	Max. 0.05 %
Cl (Chloride)	Max. 0.5 ppm
Al (Aluminium)	Max. 0.1 ppm
B (Boron)	Max. 0.08 ppm
Ba (Barium)	Max. 0.05 ppm
Ca (Calcium)	Max. 0.8 ppm
Cd (Cadmium)	Max. 0.01 ppm
Co (Cobalt)	Max. 0.01 ppm

Cr (Chromium)	Max. 0.02 ppm
Cu (Copper)	Max. 0.01 ppm
Fe (Iron)	Max. 0.1 ppm
K (Potassium)	Max. 0.1 ppm
Mg (Magnesium)	Max. 0.05 ppm
Mn (Manganese)	Max. 0.01 ppm
Na (Sodium)	Max. 0.7 ppm
Ni (Nickel)	Max. 0.01 ppm
Pb (Lead)	Max. 0.01 ppm
Sn (Tin)	Max. 0.1 ppm
Sr (Strontium)	Max. 0.02 ppm
Zn (Zinc)	Max. 0.2 ppm
Absorbance (225 nm)	Max. 0.17
Transmittance (210 nm)	Min. 20 %
Transmittance (220 nm)	Min. 50 %
Transmittance (225 nm)	Min. 68 %
Transmittance (250 nm)	Min. 95 %
Conforms to ACS	Passes test
Conforms Ph.Eur. R 1053200	Passes test
Conforms Ph.Eur. R1 1053201	Passes test
Conforms Ph.Eur. R2 1053202	Passes test

Acetonitrile ≥99.5%, AnalaR NORMAPUR® ACS, Reag. Ph. Eur. analytical reagent (supplier vwr)

Specification Test Results

Assay (on anhydrous substance)	Min. 99.5 %
IR Spectrum	Passes test
Solution (100 g/l)	Passes test
Acidity	Max. 0.0002 meq/g
Alkalinity	Max. 0.0006 meq/g

Boiling point	80 - 82 °C
Colouration	Max. 10 APHA
Density (20/4)	0.781 - 0.784
Density (20/20)	0.782 - 0.785
n 20/D	1.343 - 1.345
Evaporation residue	Max. 10 ppm
Water	Max. 0.15 %
CN (Cyanide)	Max. 50 ppm
Cd (Cadmium)	Max. 0.02 ppm
Co (Cobalt)	Max. 0.01 ppm
Cu (Copper)	Max. 0.04 ppm
Fe (Iron)	Max. 0.05 ppm
Mn (Manganese)	Max. 0.01 ppm
Ni (Nickel)	Max. 0.02 ppm
Pb (Lead)	Max. 0.05 ppm
Zn (Zinc)	Max. 0.1 ppm
Transmittance (255-420 nm)	Min. 98 %
Conforms to ACS	Passes test
Conforms to Reag. Ph.Eur.	Passes test
